# Education Research: Feasibility, Reliability and Educational Value of Neuromuscular Ultrasound Training in a Canadian Neurology Residency Program: A Pilot Study

**DOI:** 10.1212/NE9.0000000000200166

**Published:** 2024-10-04

**Authors:** Ahmed N. Alzaabi, Gurpreet Chaggar, Mohammed Wasif Hussain, Vijay J. Daniels, Grayson Beecher

**Affiliations:** From the Division of Neurology, Department of Medicine (A.N.A., G.C., M.W.H., G.B.), Department of Medicine (V.J.D.), and Neuroscience and Mental Health Institute (NMHI) (G.B.), University of Alberta, Edmonton, Canada.

## Abstract

**Background and Objectives:**

Point-of-care neuromuscular ultrasound (NMUS) is increasingly used in the evaluation of peripheral nervous system disorders; however, there remains a gap in education and training for neurology residents. We evaluated whether neurology residents can feasibly be trained in basic NMUS skills and nerve cross-sectional area (CSA) measurement and whether they value incorporation of this training into their curriculum.

**Methods:**

Participants included neurology residents (postgraduate years 1–5) at the University of Alberta (Edmonton, Alberta, Canada). All completed pretraining and posttraining surveys using a Likert scale, rating their confidence in independently performing NMUS and their degree of agreement regarding the educational value of NMUS training. Residents underwent training (7 hours) comprising 1 didactic and 2 hands-on sessions, detailing NMUS of median, ulnar, and fibular nerves. Participants could then opt-in to a posttraining testing session where CSA measurements (mm^2^) of the median, ulnar, and fibular nerve at multiple sites were independently performed on 3 healthy volunteers and compared with measurements obtained by the trainer.

**Results:**

Eighteen residents participated in training and pretraining/posttraining surveys. Nine completed the testing component. Nerve CSA measurement reliability between the trainer and trainees across all nerve sites combined was very good (intraclass correlation coefficient [ICC] 0.93, 95% CI 0.83–0.96) but varied by nerve and site. ICC was good to very good (0.62–0.95) except for the ulnar nerve-distal forearm/wrist (0.39–0.58) and fibular nerve-fibular head (0.12) sites. The coefficient of variation (CoV) across all sites was 19.6% (95% CI 17.3–21.8) and best for the median nerve-wrist site at 15.5% (9.8–20.8). The mean absolute difference between trainer and trainee measurements was low (<0.5 mm^2^ across all sites). Comparing pretraining and posttraining survey responses, there was a significant increase in agreement that basic NMUS operational skills were obtained and in confidence in independently measuring each nerve. NMUS training was considered a valuable component of a neurology residency program curriculum (median rating: strongly agree).

**Discussion:**

Neurology residents across stages of training can acquire basic NMUS and CSA measurement skills of the median and ulnar nerves after 2 half-days of training and value incorporation of NMUS training in their educational curriculum.

## Introduction

Point-of-care neuromuscular ultrasound (NMUS) is a useful tool in the evaluation of patients with disorders of peripheral nerve and muscle, which affects how these conditions are diagnosed and managed.^1,2^ This modality offers greater spatial resolution at lower cost than MRI; allows for dynamic evaluation of the nerve and muscle; and is painless, noninvasive, and portable.^3^ NMUS has demonstrated diagnostic use in the evaluation of common neurologic presentations including mononeuropathies (e.g., carpal tunnel syndrome and ulnar neuropathy at the elbow),^4,5^ traumatic nerve injuries,^6^ inflammatory and hereditary neuropathies,^7-11^ motor neuron diseases,^12^ myopathies,^13^ and diaphragmatic weakness,^14^ among others.^3^ As a testament to its value when used as an adjunct to the neurologic and electrodiagnostic evaluation, NMUS is now incorporated in diagnostic guidelines for carpal tunnel syndrome and chronic inflammatory demyelinating polyneuropathy.^1,15-18^ Despite the advantages and demonstrated utility of NMUS, it is not widely adopted in clinical practice. A major factor limiting its uptake is an educational gap in its use, stemming from the absence of an established curriculum and the limited training available for neurology trainees outside of highly specialized tertiary academic centers.^19^

Point-of-care ultrasound education is becoming an increasingly common component of Canadian undergraduate and postgraduate medical education, owing to its applicability across multiple medical disciplines and the value placed on it by both program administrators and trainees themselves.^20-25^ As the clinical applicability of NMUS broadens and its use becomes more commonplace, it is important to evaluate whether neurology residents would similarly value the incorporation of basic NMUS teaching into their postgraduate medical education curriculum. Whether such training is feasible in a neurology residency program and whether neurology residents can achieve basic competency in NMUS skills over short periods of training also remain uncertain because real-time NMUS image acquisition by neurology residents has not been evaluated. Previous NMUS studies evaluating the ability of postgraduate trainees to accurately perform nerve or muscle ultrasound are limited to incorporation of individual neuromuscular fellows^26^ and orthopaedic residents and fellows^27^ with varying levels of prestudy NMUS experience but have suggested that NMUS skills can be reliably obtained over relatively short periods of supervised training. While these studies demonstrate that NMUS training could potentially be incorporated into the neuromuscular education curricula for neurology residents, what is not well known is the value residents place on this training, which is important for uptake.

In this pilot study, we evaluated whether neurology residents can feasibly be trained in basic NMUS of commonly evaluated nerves over a short period of time, by assessing their ability to independently and accurately measure the cross-sectional area (CSA) of 3 peripheral nerves (median, ulnar, and fibular) at multiple sites compared with a neuromuscular subspecialist trainer with experience in NMUS. We further evaluated whether residents reported improvement in their basic NMUS operational knowledge and skills in CSA measurement of individual nerves and whether they felt NMUS training would be a valuable addition to a neurology residency education curriculum through pretraining and posttraining surveys.

## Methods

### Standard Protocol Approvals, Registrations, and Participant Consents

This study was approved by the University of Alberta Research Ethics Board. All participants provided informed, written consent before enrollment.

### Study Design and Data Collection

Adult neurology residents from the University of Alberta (postgraduate years [PGYs] 1–5) were recruited to participate. The study consisted of pretraining and posttraining survey questionnaires, a mandatory training component, and an optional testing component. The pretraining questionnaire was completed immediately before the first training session, and the posttraining questionnaire was completed 1 week after the last training session, just before the testing session. The number of hours of pretraining general ultrasound experience was recorded for each participant. Questions on the pretraining and posttraining questionnaires (Tables 1–3) were derived from expert consensus criteria on knowledge and skills that should be acquired to achieve minimal competency in NMUS and expert-proposed guidelines for basic NMUS training.^28,29^ Survey questions assessed residents' subjective grading of their understanding of basic ultrasound and NMUS principles on a Likert scale rating the degree of agreement (1 = strongly disagree to 5 = strongly agree) and their confidence in independently performing NMUS on a Likert scale rating degree of confidence (1 = not at all confident to 5 completely confident). The posttraining survey included additional questions about participants' subjective feelings on educational value and enjoyment of the NMUS training.

**Table 1:** Pretraining and Posttraining Self-Reported Participant Ratings of Basic Neuromuscular Ultrasound Knowledge and Operational Skills

Survey question	Pretraining score		Posttraining score		*p* Value
	Median	IQR	Median	IQR	
1. I have a basic understanding of ultrasound physics	3	2–3	4	3–4	0.001
2. I have a basic understanding of the indications and limitations of neuromuscular ultrasound	2	2–3	4	4–4	0.0002
3. I have a basic understanding of common ultrasound artifacts	2	2–3	4	4–4	0.0002
4. I can confidently identify the normal sonographic appearance of nerve, muscle, tendon, bone, and blood vessels	1	1–2	4	3–4	0.0003
5. I can confidently differentiate normal vs abnormal nerve echogenicity, size (cross-sectional area, diameter), mobility, and vascularity	1	1–1	3	3–4	0.0002
6. I can confidently independently turn on the ultrasound machine and begin an examination	2	1–3	4	4–5	0.0003
7. I can confidently adjust the gain of an ultrasound image to improve image quality	1	1–3	4	4–4	0.0003
8. I can confidently adjust the depth and focal point of an ultrasound image to improve image quality	1	1–2	4	4–5	0.0003
9. I can confidently handle an ultrasound probe to obtain a clear image	2	1–4	4	4–4	0.0009
10. I can confidently measure nerve cross-sectional area	1	1–1	4	4–4	0.0002

Abbreviation: IQR = interquartile range.

Questions 1–3 are rated on a Likert scale rating degree of agreement (1 = strongly disagree, 2 = disagree, 3 = somewhat agree, 4 = agree, 5 = strongly agree). Questions 4–10 are rated on a Likert scale rating degree of confidence (1 = not at all confident, 2 = slightly confident, 3 = somewhat confident, 4 = mostly confident, 5 = completely confident).

**Table 2:** Pretraining and Posttraining Self-Reported Participant Ratings of Confidence in Neuromuscular Ultrasound of the Median, Ulnar and Fibular Nerves

Survey question	Pretraining score		Posttraining score		
	Median	IQR	Median	IQR	*p* Value
11. I can confidently measure the cross-sectional area of the median nerve at the wrist	1	1–1	4	4–4	0.0002
12. I can confidently measure the cross-sectional area of the median nerve at the forearm	1	1–1	4	4–4	0.0002
13. I can confidently measure the cross-sectional area of the median nerve at the mid-humerus	1	1–1	4	4–4	0.0002
14. I can confidently measure the cross-sectional area of the ulnar nerve at the wrist	1	1–1	4	3–4	0.0002
15. I can confidently measure the cross-sectional area of the ulnar nerve at the elbow	1	1–1	3.5	3–4	0.0002
16. I can confidently measure the cross-sectional area of the ulnar nerve at the mid-humerus	1	1–1	4	3–4	0.0002
17. I can confidently measure the cross-sectional area of the fibular nerve at the fibular head	1	1–1	4	3–4	0.0002

Abbreviation: IQR = interquartile range.

Questions are rated on a Likert scale rating degree of confidence (1 = not at all confident, 2 = slightly confident, 3 = somewhat confident, 4 = mostly confident, 5 = completely confident).

**Table 3:** Posttraining Self-Reported Participant Rating of Neuromuscular Ultrasound Training Value

Survey question	Posttraining score	
	Median	IQR
18. The neuromuscular ultrasound training sessions were enjoyable	5	4–5
19. The neuromuscular ultrasound training sessions were effective in teaching me basic ultrasound skills	5	4–5
20. I improved my skills in nerve ultrasound as a result of the training sessions	5	4–5
21. I would like to learn how to examine other peripheral nerves using ultrasound	5	4–5
22. The neuromuscular ultrasound training sessions would be a valuable component of a neurology residency program curriculum	5	5–5

Abbreviation: IQR = interquartile range.

Questions are rated on a Likert scale rating degree of agreement (1 = strongly disagree, 2 = disagree, 3 = somewhat agree, 4 = agree, 5 = strongly agree).

All training sessions were provided by a neuromuscular fellowship-trained neurologist with 4 years of NMUS experience (G.B.). The training component consisted of a 1-hour didactic session on the basics of ultrasound operation; NMUS principles; scanning approaches for the median, ulnar, and fibular nerves; and 2 3-hour hands-on NMUS sessions each 1 week apart. Ultrasound demonstration and training were performed using a high-resolution portable ultrasound system (Sonosite PX; Fujifilm Sonosite, Toronto, ON, Canada) with a 4–15-MHz linear array transducer. Three machines were available for dedicated trainee use. The hands-on sessions began with a demonstration of median, ulnar, and fibular nerve ultrasound by the trainer on a healthy volunteer. These nerves were chosen as scanning protocols for common focal neuropathies (including carpal tunnel syndrome, ulnar neuropathy at the elbow and wrist, and fibular neuropathy) that achieved unanimous consensus as skills that should be acquired to achieve minimal competency in NMUS, per recent expert consensus criteria.^28^ The trainer demonstrated how to obtain CSA measurements by tracing the nerve just inside the hyperechoic epineurium, at the following sites: median (wrist, distal forearm, and mid-humerus), ulnar (wrist, distal forearm, below elbow, elbow, above elbow, and mid-humerus), and fibular (adjacent to the fibular head).

After the training sessions, participants were given the option of participating in an NMUS testing session where they would independently perform ultrasound and record CSA measurements (mm^2^) of the median, ulnar, and fibular nerves at the prespecified sites mentioned above, on 3 healthy volunteers different from those who volunteered in the training sessions. The trainer completed CSA measurements from each volunteer to serve as the reference standard before resident participants completed the measurements independently. Single measurements were taken from each nerve site without averaging, in line with clinical practice. Trainee images and CSA measurements were reviewed and recorded by the trainer.

### Statistics

Data were analyzed, and descriptive statistics were obtained with IBM SPSS statistics (IBM SPSS Statistics for Macintosh, version 29.0, 2022; Armonk, NY). The Wilcoxon matched-pair signed-rank test was used to compare the difference between pretraining and posttraining survey scores. A Bonferroni correction was applied for multiple comparisons, with a *p* value <0.003 considered statistically significant. Inter-rater reliability of trainer and trainee CSA measurements at each nerve site was assessed using the intraclass correlation coefficient (ICC) to determine the degree of absolute agreement: <0.21 (poor), 0.21–0.40 (fair), 0.41–0.60 (moderate), 0.61–0.80 (good), and 0.81–1.00 (very good). Mean CSA and absolute trainer-trainee difference between CSA measurements were calculated. The coefficient of variation of CSA measurements for each nerve site was determined by (standard deviation of the mean absolute trainer-trainee CSA differences/mean CSA) × 100%, as previously described.^26^

### Data Availability

Anonymized data not published within this article will be made available by request from any qualified investigator.

## Results

### Study Participants

Eighteen (72%) of 25 adult neurology residents (with representation across all 5 years of training; PGY1–3, PGY2–4, PGY3–6, PGY4–4, PGY5–1) participated in the study and completed the pretraining and posttraining survey questionnaires. Compared with the residency as a whole at the time of the study, where there were 5 residents per year in training, save for the PGY3 cohort where there were 6 residents, there was lesser participation from PGY5 residents. Only the PGY4–5 residents had completed a neuromuscular or EMG clinical rotation before the study, and no resident was completing a rotation during the study period. General ultrasound experience was reported in 14 participants while 4 had none (median experience: 3 hours; range 0–8 hours). Of these, 9 residents (50%; PGY1–1, PGY2–1, PGY3–3, PGY4–3, PGY5–1) were able to participate in the NMUS testing session.

### Participant Survey Results

Participant responses to pretraining and posttraining survey questionnaires are listed in Tables 1–3. Comparing pretraining and posttraining survey responses, there was a significant (all *p* < 0.0001) increase in self-report that basic NMUS operational skills were obtained (Table 1) and in confidence of ability to measure median, ulnar, and fibular nerves at specified sites (Table 2). Median agreement with knowledge of basic ultrasound operational skills (Table 1) improved from 2 (disagree) to 4 (agree) after the training sessions. Participant confidence in measuring the median, ulnar, and fibular nerves improved from a baseline median of 1 (not at all confident) to 4 (mostly confident) for each individual site, save for the ulnar nerve at the elbow which improved to a median of 3.5 (Table 2).

All participants agreed or strongly agreed that the training sessions were enjoyable, effective, and improved their ultrasound skills (Table 3). In addition, all participants somewhat to strongly agreed that NMUS would be a valuable component of a neurology residency program curriculum (median 5, strongly agree, IQR 5–5).

### Evaluation of Nerve CSA Measurements by Trainees

Nine participants took part in the posttraining nerve CSA testing session. Nerve CSA measurements between the trainer and trainees showed very good reliability overall, with an ICC of 0.93 (95% CI 0.83–0.96) for all nerve site measurements combined. There was variability depending on the nerve and site examined (Table 4). The ICC for the median nerve was very good at all sites (all ≥0.87). However, individual ICC values for the ulnar and fibular nerves were generally lower than that for the median nerve. Specifically, ICC values for the ulnar nerve ranged from good to very good spanning the below elbow (0.62, 95% CI 0.38–0.91) to the mid-humerus sites (0.95, 95% CI 0.77–0.99), to moderate at the wrist (0.58, 95% CI 0.35–0.89) and fair at the distal forearm site (0.39, 95% CI 0.28–0.87). For the fibular nerve, the ICC was poor at 0.12 (95% CI 0.09–0.81).

**Table 4:** Nerve CSA Measurements and Reliability Comparing Trainer and Trainees

Nerve site	Nerve CSA (mm^2^)				ICC (95% CI)	%CoV (95% CI)
	Trainer Mean (SD)	Trainees Mean (SD)	Range	Mean difference (SD)		
Median nerve at wrist	7.7 (1.2)	7.2 (1.2)	5.0–9.0	0.5 (1.1)	0.87 (0.40–0.99)	15.5 (9.8–20.8)
Median nerve at forearm	7.0 (1.0)	6.9 (1.6)	4.0–10.0	0.1 (1.4)	0.88 (0.52–0.97)	21.2 (13.6–28.9)
Median nerve at mid-arm	8.7 (1.5)	8.1 (1.9)	5.0–10.0	0.5 (1.3)	0.92 (0.65–0.98)	16.7 (10.7–22.8)
Ulnar nerve at wrist	4.7 (0.6)	4.4 (1.0)	3.0–6.0	0.2 (0.9)	0.58 (0.35–0.89)	22.5 (14.4–30.7)
Ulnar nerve at distal forearm	4.7 (0.6)	4.9 (1.0)	3.0–7.0	−0.1 (0.9)	0.39 (0.28–0.87)	19.9 (12.8–27.3)
Ulnar nerve below elbow	6.3 (1.5)	6.1 (1.2)	3.0–8.0	0.1 (1.5)	0.62 (0.38–0.91)	25.0 (15.9–34.2)
Ulnar nerve at elbow	7.0 (1.0)	6.9 (1.5)	5.0–10.0	0.1 (1.3)	0.76 (0.41–0.95)	19.8 (12.7–26.9)
Ulnar nerve above elbow	6.0 (1.0)	6.5 (1.2)	4.0–9.0	0.1 (1.3)	0.84 (0.26–0.96)	16.2 (10.4–22.1)
Ulnar nerve at mid-arm	5.7 (1.5)	5.9 (1.5)	4.0–9.0	−0.5 (1.0)	0.95 (0.77–0.99)	17.2 (11.1–23.5)
Fibular nerve at fibular head	8.0 (1.5)	8.0 (1.7)	5.0–11.0	−0.2 (1.0)	0.12 (0.09–0.81)	21.1 (13.5–28.8)

Abbreviations: CoV = coefficient of variation; CSA = cross-sectional area; ICC = intraclass correlation coefficient.

Mean absolute differences between trainer and trainee nerve CSA measurements were generally low at ≤0.5 mm^2^ across all sites (Table 4). The average CoV across all nerve sites from each volunteer, as measured by each trainee and the trainer, was 19.6% (95% CI 17.3–21.8). Average values were lower for the median than ulnar and fibular nerve sites and ranged from 15.5% (95% CI 9.8–20.8) for the median nerve at the wrist to 25.0% (95% CI 15.9–34.2) for the ulnar nerve below the elbow.

## Discussion

This study demonstrates that Canadian neurology residents at various stages of training with minimal experience with ultrasound and none in NMUS are able to acquire basic skills of independently performing NMUS and measuring CSA in clinically relevant peripheral nerves with 2 half-days of training. Pretraining and posttraining questionnaires highlight that neurology residents showed improvement in their level of confidence in NMUS performance over a short period of training and also place high value on NMUS training itself. NMUS training seems feasible within neurology residency training curricula and should be incorporated based on its evolving clinical utility and educational value as perceived by residents themselves.

Measuring nerve CSA using ultrasound is a valid and reliable method of assessing peripheral nerves.^30^ However, our study aimed to determine whether neurology residents specifically could be trained to independently and accurately perform NMUS and measure CSA of commonly evaluated peripheral nerves in real time. Previous studies evaluating trainer-trainee inter-rater reliability of real-time CSA measurements have demonstrated relatively high reliability after limited training, however having not involved neurology residents. Our study's overall ICC of 0.93 and CoV of 19.6% is similar to a prior study^26^ that found very good inter-rater reliability (ICC = 0.88) and CoV of 17% for upper limb nerve CSA measurements between an expert with greater than 10 years of experience in NMUS and 2 neuromuscular fellow trainees with either 2 or 12 months of supervised training. This previous retrospective study examined 42 patients referred for upper limb ultrasound, including those with and without pathology. In line with our results, the median nerve demonstrated the greatest inter-rater reliability overall (ICC = 0.88 in both studies) and the median nerve at the wrist demonstrated the lowest variance (%CoV 9.33 vs 15.5 in our study). Lower variability and ICC values above 0.80 for the median nerve at the wrist are important to emphasize, given that carpal tunnel syndrome evaluation is one of the most common indications for NMUS and competency in this assessment is clinically relevant.^31^ Indeed, in the prior study,^26^ 25 of 42 patients evaluated had NMUS features compatible with carpal tunnel syndrome. While increased training experience can reduce CSA measurement variability, the novice trainee with 2 months of experience in that study demonstrated similar variability in nerve CSA measurements across the same nerve sites examined in our study (%CoV range 13.12–31.04 vs 15.5–25.0 in our study) where trainees had less experience (2 half-days total). Mean differences in nerve CSA measurements between the trainer and trainees in our study were importantly low (≤0.5 mm^2^ across all sites), unlikely to produce clinically meaningful differences in CSA reporting based on the average expected CSA for the nerves evaluated.^32-35^

While neurology residents have not been incorporated in studies of real-time NMUS image acquisition and CSA measurement, 1 study did incorporate a neurology resident, evaluating the reliability of nerve CSA and muscle thickness measurements performed on provided still images.^30^ This study included muscle and nerve ultrasound images from 4 healthy volunteers with measurements independently performed by 5 participants with varying NMUS experience: a neurology resident (less than 3 months), clinical neurophysiology fellow (6 months), faculty neurologist (2 years), clinical neurophysiology technician (5 years), and another faculty neurologist (10 years). Overall, intra-rater and inter-rater reliability for tracing nerve CSA with built-in ultrasound calipers was high, although intra-rater correlation coefficients demonstrated the greatest range in the neurology resident (0.174–0.966). Real-time image acquisition and nerve CSA measurement were not performed by the neurology resident; however, the overall high inter-rater reliability observed across 9 neurology residents in our study suggests that this skill can be acquired in this trainee group.

The optimal duration of NMUS experience and the number of healthy individuals and patients evaluated to achieve basic levels of competency remain uncertain.^28,36^ In this study, after 2 half-days of predominantly hands-on training, neurology residents demonstrated overall high inter-rater reliability in nerve CSA measurements in 3 healthy volunteers across median and ulnar nerve sites assessed, but poor reliability at the fibular nerve site, suggesting that the latter site may require additional experience to achieve basic competency in normal trainees. Trainees in this study demonstrated difficulty in maintaining the probe both in plane with and perpendicular to the fibular nerve in the area where it winds toward and runs adjacent to the fibular head, which may have contributed to the low ICC value obtained and the higher variability in measurement compared with the trainer (CoV 21.1%). Shorter durations of NMUS training than those used in our study may be applicable for individual nerve sites, depending on the indication for NMUS. Indeed, moderate inter-rater reliability (ICC = 0.59) in measuring the median nerve CSA at the wrist in 11 healthy volunteers was observed across a first-year orthopaedic resident with no NMUS training, an orthopaedic hand fellow with limited training, and a staff orthopaedic surgeon with extensive training, who all underwent a short, 30-minute training session before commencing measurements.^27^ Our study substantiates that NMUS trainees can reliably use NMUS to specifically assess peripheral nerve CSA in the median and ulnar nerves with limited (2 half-days) previous hands-on training.

It is important to note that, as evidenced by the pretraining and posttraining questionnaire results, neurology residents in our study placed high value on the incorporation of NMUS in their residency curriculum and felt that the training sessions improved their NMUS skills. With ultrasound being a readily available tool and the clear capability of beginner sonographers to acquire the technical skills to reliably measure CSA of common peripheral nerves in real time, the lack of a dedicated curriculum and local expertise rather than the acquisition of technical skills in inexperienced sonographers seem to be the barrier to integrating NMUS into neurology residency training. A general consensus statement for guidelines regarding NMUS training outlined that “structured basic neuromuscular ultrasound training is recommended for all physicians who practice electrodiagnosis,” a statement that is especially relevant to neurology residents who will receive training in performing and interpreting electrodiagnostic studies and fellows training in neuromuscular neurology.^29^ The experts providing these consensus guidelines found difficulty in standardizing the total hours of didactic and hands-on learning to establish competency, although studies such as ours could be used as a frame of reference for hours required to produce reliability of nerve CSA measurement, for at least the median and ulnar nerves.

The optimal method of delivery of NMUS education in a neurology residency program is uncertain, particularly when other educational topics compete for time. A component of didactic learning is certainly required; however, 50%–75% of time is recommended for hands-on training in NMUS courses.^29^ A crucial barrier to obtaining adequate hands-on training time is the lack of local expertise in NMUS and availability of resources for training (i.e., access to ultrasound equipment and educational material), particularly in Canada where NMUS is not routinely performed by neurologists and where a pathway to certification of competency in NMUS does not yet exist. Virtual or asynchronous courses such as those used during the coronavirus disease 2019 pandemic can help bridge the gap in NMUS education.^37^ However, delivery of hands-on training in real-time image acquisition and measurement, either locally or through external courses, is still necessary.

This study has limitations, including those inherent to a single-center study such as smaller recruitment numbers affecting effect size, limited generalizability across centers, selection and institutional bias, and lack of external validation. Only 9 residents were able to complete the posttraining nerve CSA testing session. Coordinating individual resident schedules such that all residents could attend the didactic, hands-on training, and posttraining testing sessions was difficult and may have affected resident participation in the posttraining session (i.e., postcall residents, those absent through vacation, or those off-site would have understandably been unable to opt in to the testing session). Delivery of the training and testing sessions over a condensed weekend or evening period, for example, may have led to higher participation overall and will be considered for future training studies. While training sessions included near-equivalent representation across years of residency (save for PGY5), the testing session group comprised mainly senior residents (PGY3–4) who may have had greater comfort with peripheral nerve anatomy, potentially contributing to more reliable CSA measurements and interest in NMUS training, affecting generalizability of the results. However, none of the PGY1–3 residents participating in the training or testing sessions had completed or were in the process of completing a dedicated neuromuscular rotation before or during the study itself. Owing to the small participant numbers, we could not meaningfully statistically assess the impact of year-in-training on the survey and CSA measurement results. The self-selected nature of the testing group also contributes to selection bias because participants interested in neuromuscular neurology may have been more likely to participate and provide higher ratings of agreement on the posttraining survey. We did not collect information regarding baseline interest in neuromuscular neurology and, therefore, cannot comment on this potential confounder. Smaller participant numbers could also contribute to a sense of a lack of anonymity and again lead participants to rate the training course higher than they would otherwise. The small number of participants evaluated in the posttraining sessions represents another limitation in sample size, which could increase variability and reduce ICC values across examiner CSA measurements for individual nerve sites. Moreover, the subjects assessed were healthy volunteers and thus we are unable to comment whether the skills acquired would definitively translate into measuring abnormal nerve CSA in patients with specific peripheral nerve disorders. Furthermore, while the period between the final hands-on training session and the testing session was 1 week, a subsequent repeat testing session at a later date was not performed, and therefore, we are unable to comment whether skills are maintained over longer periods. However, NMUS training studies available have established good intra-rater reliability.^27,28,38^

This study combined intervention variables including time, didactic learning, and hands-on sessions; therefore, without a control group or crossover, we could not minimize confounding effects of cointervention, which makes it difficult to determine which specific component of training was responsible for the results. Last, another factor to consider when interpreting the results is the effectiveness of the trainer as an educator. The quality of professional development of educators and their instruction delivery affects the achievement of learners and contributes to variance in learner achievement observed between educators.^39,40^ This study was conducted with a single trainer, and thus, results may be confounded by the effectiveness of the teacher.

In conclusion, this pilot study demonstrates that neurology residents find value in the incorporation of NMUS in residency training and confidence in their ability to perform basic peripheral nerve ultrasound was increased greatly after a short training period. Objectively, the real-time measurement of nerve CSA in the upper limbs demonstrated good-to–very good inter-rater reliability, which was not seen in the fibular nerve across the fibular head. Residency seems an appropriate time to introduce NMUS principles and provide training in assessment of common peripheral nerves, which can help reinforce peripheral neuroanatomy concepts and may also teach general ultrasound skills that can be used in other applications (e.g., imaging carotid arteries and guiding interventional procedures). Future studies could further assess inter-rater reliability by evaluating the residents' ability to measure nerve CSA in multiple healthy controls and in patients with peripheral nervous system disorders. Additional nerve and/or muscle sites could also be added to the evaluation to assess for varying degrees of complexity in NMUS. The addition of repeated, spaced assessment with varying amount of time from the final training session could assess the maintenance and reproducibility in reliability over time.

## Appendix Authors

**Table:**

Name	Location	Contribution
Ahmed N. Alzaabi, MD	Department of Medicine, University of Alberta, Edmonton, Canada	Drafting/revision of the manuscript for content, including medical writing for content; analysis or interpretation of data
Gurpreet Chaggar, MD	Department of Medicine, University of Alberta, Edmonton, Canada	Drafting/revision of the manuscript for content, including medical writing for content; analysis or interpretation of data
Mohammed Wasif Hussain, MBBS	Department of Medicine, University of Alberta, Edmonton, Canada	Drafting/revision of the manuscript for content, including medical writing for content; study concept or design
Vijay J. Daniels, MD, MHPE	Department of Medicine, University of Alberta, Edmonton, Canada	Drafting/revision of the manuscript for content, including medical writing for content; study concept or design
Grayson Beecher, MD	Division of Neurology, Department of Medicine, and Neuroscience and Mental Health Institute (NMHI), University of Alberta, Edmonton, Canada	Drafting/revision of the manuscript for content, including medical writing for content; major role in the acquisition of data; study concept or design; analysis or interpretation of data

## Glossary

CoV: coefficient of variation
CSA: cross-sectional area
ICC: intraclass correlation coefficient
IQR: interquartile range
NMUS: neuromuscular ultrasound
PGY: postgraduate year
